# Changes of the Aroma Composition and Other Quality Traits of Blueberry ‘Garden Blue’ during the Cold Storage and Subsequent Shelf Life

**DOI:** 10.3390/foods9091223

**Published:** 2020-09-02

**Authors:** Xiaoxue Yan, Jun Yan, Siyi Pan, Fang Yuan

**Affiliations:** 1 College of Food Science and Technology, Huazhong Agricultural University, Wuhan 430070, China; yxx1647883233@163.com (X.Y.); juneyan19@163.com (J.Y.); pansiyi@mail.hzau.edu.cn (S.P.); 2 Key Laboratory of Environment Correlative Dietology, Huazhong Agricultural University, Wuhan 430070, China

**Keywords:** rabbiteye blueberry, postharvest storage, firmness, aroma compounds, off-odor

## Abstract

The changes of volatile composition and other quality traits of blueberry during postharvest storage were investigated. Blueberries were packaged in vented clam-shell containers, and stored at 0 °C for 0, 15 and 60 days, followed by storage at room temperature (25 °C) for up to 8 days for quality evaluation. The firmness, pH, and total soluble solids increased by 8.42%, 8.92% and 42.9%, respectively, after 60 days of storage at 0 °C. Titratable acidity decreased 18.1% after 60 days of storage at 0 °C. The volatile change was monitored using headspace–solid-phase microextraction–gas chromatography–quadrupole time-of-flight–mass spectrometry (HS-SPME-TOF-MS) and off-odor was evaluated by sensory panel. Volatile compounds generally showed a downward trend during cold storage. However, the subsequent shelf life was the most remarkable period of volatile change, and was represented by the strong fluctuation of ethyl acetate and the rapid decrease of terpenoids. Extending storage from 15 to 60 days under cold condition still resulted in an acceptable odor. However, subsequent storage at higher temperature resulted in a quick deterioration in sensory acceptability. The results proved that cold storage was a reliable way to maintain the quality of blueberry, and flavor deterioration during subsequent shelf life was more fatal to the blueberry flavor.

## 1. Introduction

Due to their unique flavor and nutritional value, blueberry fruit and products are sold at a high price in the international market, and the production is growing every year [1,2]. As the production increases, blueberry deterioration is often a problem for the industry. Like other soft berries, blueberries can be processed into blueberry juice, jam and other products, but the market price is several times lower than that of fresh fruit. Therefore, the sale of fresh fruit is important for the blueberry industry. Thanks to rapidly developed preservation technology, the shelf life of blueberries can be extended to up to one month [3,4,5,6]. Cold storage and transportation are most the widely used means of blueberry preservation all over the world [7]. However, for countries with an undeveloped cold chain system, room temperature is still a common condition for short-distance transportation and retail display, which may lead to problems such as the loss of aroma and the generation of off-odor, which can greatly affect the flavor perception of consumers.

The aroma of small berries is more complicated than other fruit and is hard to manipulate. Unlike the climacteric fruit, ethylene does not stimulate the ripening of small berries such as strawberries [8]. Blueberries are climacteric fruits and will respond to ethylene. However, blueberry flavor does not improve by ethylene after harvest [9]. Therefore, a comprehensive understanding of the berry aroma change is essential for possible flavor manipulation during postharvest storage. Among the small berries, strawberry and raspberry are the most widely studied in aroma research [10,11]. More than 360 and 200 volatile compounds have been reported in strawberries and raspberries, respectively [12]. Relatively speaking, the research on the aroma of the blueberry is lagging. There have been fewer than 100 volatile compounds reported in blueberry [13]. The main aroma substances found in rabbiteye blueberry are ethyl acetate, limonene, hexanol, (Z)-3-hexenol, heptanol, β-ionone, terpinene-4-ol, α-terpineol, vanillin, nerol and eugenol [14,15]. Farneti et al. [16] analyzed the volatile composition of eleven different blueberry cultivars and found that for most cultivars, aldehydes, alcohols, terpenoids, and esters can be used as putative biomarkers to evaluate the blueberry aroma variations. Recent reports on blueberry wine and vinegar also showed that terpenes such as linalool and α-terpineol were important berry-derived aroma compounds in blueberries [17,18]. Zhu et al. [19] reported that esters, aldehydes, C13-norisoprenoids, as well as several terpenes and eugenol, were the aroma-active compounds in freshly pressed blueberry juice. From the existing reports, the main aroma substances of fresh blueberry fruits are alcohols, esters, terpenes and aldehydes, and the aroma contents are affected by cultivar and environmental factors [16,20,21,22].

The methods used for blueberry volatile analysis were thoroughly reviewed by Sater et al. [2]. Static headspace sampling using solid phase microextraction (SPME) is the most commonly used extraction method in the blueberry volatile studies. The separation of compounds in blueberry studies is most commonly accomplished by a gas chromatograph (GC) directly connected to a mass spectrometer (MS). Older GCs often use a flame ionized detector (FID) to quantify the relative abundance of each compound. In recent years, more sensitive methods such as headspace–solid-phase microextraction–gas chromatography–quadrupole time-of-flight–mass spectrometry (HS-SPME-TOF-MS) and proton transfer reaction–time-of-flight-mass spectrometry (PTR-TOF-MS) technology have been applied to blueberry volatile analysis [16,23].

In the postharvest chain, flavor maintenance is an important aspect for ensuring the quality of fresh fruit. Many studies have been carried out in blueberry for understanding the impact of different storage conditions and treatments with regard to phenolics, anthocyanins and flavonoids [24,25]. However, there are fewer reports about ways to preserve the aroma/flavor quality of blueberry. Based on our literature search, only one study showed that the content of volatile terpenes, phenols and anthocyanins in blueberry fruits was increased by UV-B irradiation during postharvest storage [26], proving that it is feasible to manipulate the aroma quality of blueberry by appropriate means. Therefore, in this study, we investigated the quality changes of blueberries during different durations of cold storage conditions and subsequent room temperature storage. The volatile composition change was monitored using HS-SPME-GC-QTOF-MS and off-odor was evaluated. The aim of this study was to have a better understanding of the changes occurring in blueberry during postharvest storage.

## 2. Materials and Methods

### 2.1. Plant Material and Postharvest Storage

Blueberries were hand harvested from a local blueberry orchard in Huangpi, Hubei, China. The fruits of rabbiteye blueberry (*Vaccinium virgatum*) cultivar ‘Garden blue’ were randomly harvested in July of 2018. Berries that were at commercial maturity (a completely blue color) were selected. The blueberries were precooled in an air-conditioned room (~20 °C) for half an hour to remove the field heat, divided into PET boxes (classic commercial “clam-shells” container with openings for ventilation, ~125 g per box) and transported to the lab. To simulate commercial storage conditions, fruits were stored in a cooler at 0 ± 0.5 °C and 85% relative humidity (RH) in the dark for up to 60 days, and were then removed and placed at room temperature (to simulate retail display conditions) at 25 ± 0.5 °C and 60% RH under normal room light for up to 8 d. At the 0th, 15th and 60th day of cold storage, 15 boxes were picked from the top, middle and bottom of the cooler and used as samples for the subsequent 25 °C storage. At the 0th, 2nd, 4th, 6th and 8th day of 25 °C storage, three boxes were picked as three biological replicates for the following analysis.

### 2.2. Measurements of Weight Loss and Decay Index

At sampling date, each box of samples was weighed to calculate the weight loss. Decay refers to berry juice leakage, visible microbial growth, or pronounced rot on berry surface. Decay index was used for decay evaluation according to Cao et al. [27] with minor modification, calculated as follows: no decay = 1; decay berries < 5% = 2; decay berries < 10% = 3; decay berries < 20% = 4; decay berries > 20% = 5.

### 2.3. Measurements of Total Soluble Solids, pH and Titratable Acidity

For each biological replicate, approximately 30 g of berries was randomly selected and crushed in liquid nitrogen. The powdered sample was thoroughly homogenized and put in a 30 °C water bath to thaw, and centrifuged at 12,000 rpm for 10 min. The clear juice obtained was used for total soluble solids (TSS) and pH measurements. TSS was measured at room temperature using a PAL-1 pocket refractometer (Atago USA, Inc., Bellevue, WA, USA). The pH of the juice was measured using a pH meter (Rex PHS-2F, Shanghai, China). Five mL of juice was diluted with 50 mL of distilled water, and the titratable acidity (TA), as g/100 g of citric acid, was measured using burette with phenolphthalein as indicator. Three measurements were made for each sample and means were used for each biological replicate.

### 2.4. Measurement of Firmness

Ten berries without decay were randomly selected from each replicate for firmness analysis. Berry firmness was assessed using a texture analyzer (TA. XT plus, Godalming, UK), with 8 mm probe diameter, across a 7 mm distance, and a measuring speed of 0.5 mm/s [28]. Each berry was measured once. The peak force (firmness, expressed as N) was calculated by the integrated software.

### 2.5. Analysis of Volatile Compounds

The analysis of volatile metabolites was previously described by Cheng et al. [23]. Briefly, for each biological replicate, 30 g of berries were randomly selected and crushed in liquid nitrogen. 10 g of blended blueberry powder was weighed and mixed with 10 mL of propyl gallate (10 mM). The sample was homogenized at 4 °C for 24 h and centrifuged (10,000 rpm, 30 min, 4 °C). The clear supernate was used for volatile compound analysis.

The volatile compound analysis was performed using the solid phase microextraction (SPME) technique coupled with gas chromatography–quadrupole time-of-flight–mass spectrometry (GC-QTOF-MS) (7200 accurate-mass, Agilent Technologies, Santa Clara, CA, USA). Two mL of juice and 8 mL of saturated saltwater were mixed in a headspace sampling vial. Ten μL internal standard (50 mg/L 4-octanol in methanol) was added. A small magnetic stir bar was added to the vial and equilibrated at 50 °C in a water bath for 15 min with stirring. After equilibration, headspace volatiles were collected on a SPME fiber (2 cm, DVB/CAR/PDMS, 50/30 μm, Supelco, Bellefonte, PA, USA). The triple phase fiber was selected because it can cover more volatiles with different molecular weight. The adsorption time was 45 min. Headspace temperature was set at 50 °C. Desorption temperature was 250 °C, and desorption time was 5 min with split ratio of 1:10.

The GC-QTOF-MS was equipped with a HP-5MS (30 m × 250 μm × 0.25 μm) column. Oven temperature program setting: 40 °C for 5 min, increased to 180 °C at 3 °C/min, hold for 1 min, increased to 250 °C at 20 °C/min, hold for 2 min. The helium flow rate was 1.2 mL/min. The interface temperature was 300 °C and ion source temperature was 250 °C. Mass spectrum data from m/z 25 to 300 were collected. The ionization voltage was 70 eV. To ensure the accuracy of the instrument, mass calibration was performed daily.

Identification of volatile compounds were performed by comparing the mass spectra with records from external databases such as NIST, HMDB, MassBank and an internal database for the wine volatiles based on the literature, and by comparing the Kovats retention indices (RI) in NIST database and published literatures. Calibration plots were constructed using authentic standards (Table S1 (Supplementary Materials)). Ten microliters of 50 mg/L 4-octanol were added to each calibration mixture (10 mL of saturated saltwater plus authentic standards). The volatile extraction and detection methods were the same as for sample analysis. Seven-level calibration plots for each volatile compound were built using the MassHunter software to quantify the volatile compounds in the blueberry samples. Peak area of target ion for each compound was plot against the peak area of the target ion of internal standard. The GC-QTOF-MS data processing was performed with MassHunter B.06.00 software (Agilent Technologies). A representative chromatogram could be found in Supplementary Materials (Figure S1 (Supplementary Materials)). The average value of three measurements was used for each biological replicate.

### 2.6. Sensory Evaluation

The sensory test was conducted prior to storage, after storage at 0 °C for 15 and 60 days, and after post-storage conditioning at 25 °C for 4 more days, respectively. Six samples (prior to storage, 0 d at 0 °C + 4 d at 25 °C, 15 d at 0 °C, 15 d at 0 °C + 4 d at 25 °C, 60 d at 0 °C, and 60 d at 0 °C + 4 d at 25 °C) were evaluated in six different sensory sections. Eighteen panelists were recruited from the campus (10 female and 8 male). Panelists’ ages ranged from 20 to 50 years. The panelists attended three half-hour training sessions prior to the test. During the training session, the panelists got familiar with the five-point scale, and the difference between fresh and stored blueberries through discussion. During the formal sensory evaluation, a set of three sensory replications, defined as “samples” for the panelists, were presented to each panelist. Each sensory replication (or “sample”) consisted of a set of 10 berries, which were placed into one 120 mL plastic soufflé cup with lids and labeled with a random three-digit code, which corresponded to the code presented in the evaluation score sheet. The panelists were asked to take at least five berries each time, chew thoroughly and rate. Off-odor was assessed according to the 1–5 scale, in which 1 was used to refer to excellent odor; 2 for good freshness odor; 3 for neither good nor bad odor; 4 for unpleasant off-odor; and 5 for extensively severe off-odor.

### 2.7. Statistical Analysis

To determine the changes of blueberry quality parameters, a one-way ANOVA was performed for each storage day, using the days of shelf-life as treatments. For volatile compounds, a one-way ANOVA was also performed for each storage day, using the days of shelf-life as treatments. For data on sensory evaluation and decay index, a non-parametric analysis was conducted. All statistical analyses were carried out using SPSS 22.0 (SPSS, Chicago, IL, USA). A post hoc range test of Tukey’s HSD (honestly significant difference) was used to identify homogeneous subsets of means that are not different from each other.

## 3. Results

### 3.1. Weight Loss and Decay Index

**Figure 1:**
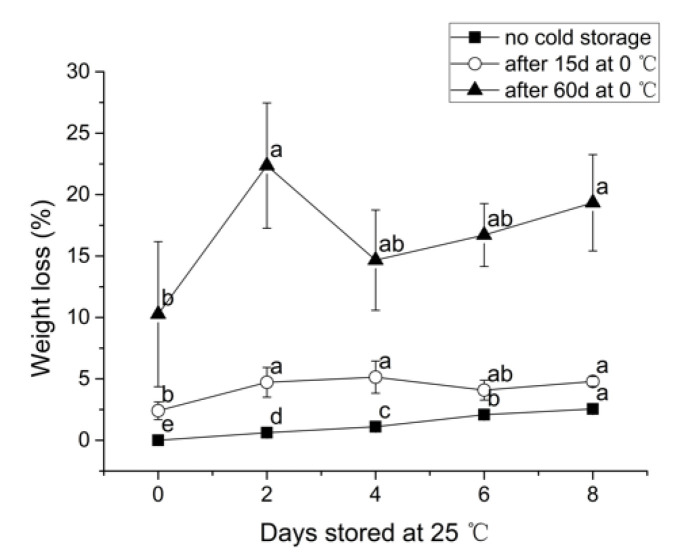
Berry weight loss during 25 °C storage period after different 0 °C storage lengths. Different letters indicate statistically significant difference (Tukey’s HSD, *p* < 0.05) between samples.

As expected, weight loss increased during storage, with longer storage time resulting in increased percentage of weight loss (Figure 1). It was unexpected that the percentage of weight loss was the highest (22.4%) after 60 d at 0 °C, followed by 2 d at 25 °C, possibly due to the abnormally high percentage of decayed berries in two of the three replicates (5.7%, 14.3% and 13.8%, respectively). We also observed a remarkably higher variation of weight loss for the samples stored at 0 °C for 60 days. Weight loss values observed at the completion of the 0 °C storage (2.4% for 15 d and 10.3% for 60 d) were similar to the values found by previous research work. Paniagua et al. [29] reported weight loss of approximately 10% after 21 d at 0 °C. Similarly, Sanford et al. [30] reported a 5.3% weight loss after storage at 0 °C for 14 d. Accordingly, the weight loss values generated in this experiment represent the range of weight loss values that are representative of possible postharvest systems.

**Figure 2:**
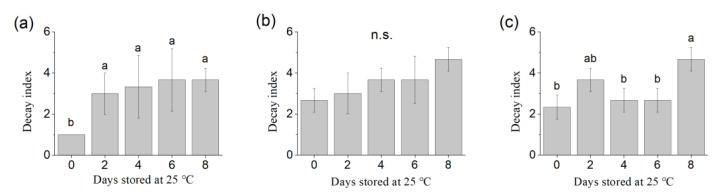
Decay index during 25 °C storage periods after (**a**) no cold storage, (**b**) 15 days of 0 °C storage and (**c**) 60 days of 0 °C storage. Different letters indicate statistically significant difference (Tukey HSD, *p* < 0.05) between samples. n.s. No statistically significant difference between samples. Decay index was calculated as follows: no decay = 1; decay berries < 5% = 2; decay berries < 10% = 3; decay berries < 20% = 4; decay berries > 20% = 5.

Decay index was calculated during the 25 °C storage period after different times of cold storage, respectively (Figure 2). The decay incidence significantly increased during the 25 °C storage period, mainly due to the juice leakage, according to our observations. Without cold storage, about 10% of the berries decayed after 8 days of storage at 25 °C. Cold storage resulted in more severe decay (about 20% of the berries decayed) at the end of the experimental period compared to the samples without cold storage. A fluctuation was observed for decay index after 60 d of cold storage due to the unexpectedly severe deterioration at the second day, possibly due to the berries being damaged during handling or temperature change. Apart from the second day, the samples stored at 25 °C for 4 and 6 days exhibited a low decay index (<3) compared to other samples (no cold and after 15 days of storage at 0 °C).

### 3.2. TSS, pH and TA

TSS of the ‘Garden blue’ was 14.3% at the time of harvest (Table 1). After 15 d and 60 d of cold storage (0 °C), the TSS increased to 14.8% and 15.7%, respectively, which is common in fruits because of the sugar concentration due to the moisture loss during the cold storage. During the 25 °C storage period, the TSS of the blueberry fruits generally showed a declining trend, indicating the sugar metabolism was activated at higher storage temperature. The pH of the berry did not change after 15 d of 0 °C storage, compared to the fresh sample at time of harvest (pH = 2.94). However, the pH increased to 3.21 after 60 d of 0 °C storage. After different cold storage times, the pH of the berry slightly increased during the 25 °C storage period for all samples, while the TA decreased.

### 3.3. Berry Firmness

For no cold storage treatment, fruit firmness significantly increased after 8 d of storage at 25 °C (approximately 29.5%) in comparison to initial firmness (4.08 N) and reached a maximum value of 5.58 N (Table 1). Firmness also showed a significant increase during the 25 °C storage period after 15 d of cold storage. For the 60 d cold storage treatment, firmness fluctuated during the 25 °C storage period due to the large sample variation, which was consistent with the weight loss and decay index data.

### 3.4. Aroma Compounds

**Figure 3:**
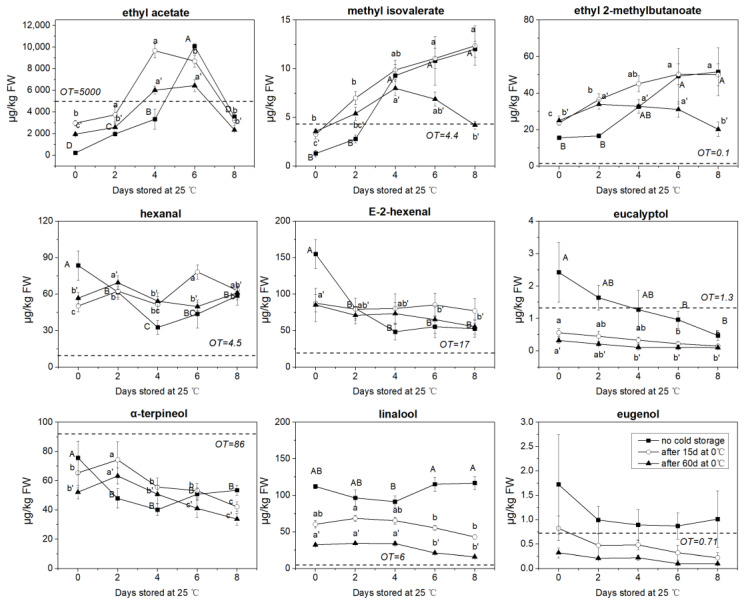
The changes of key aroma compounds in blueberry during 25 °C storage period after different lengths of 0 °C storage. Volatile concentrations are presented as µg/kg fresh weight (FW). Different letters indicate statistically significant difference (Tukey HSD, *p* < 0.05) between samples. The dashed line indicates the odor threshold (OT, μg/L in water) of the compound. The odor thresholds (μg/L in water) were obtained from [29]. Odor descriptions for each compound can be found in Table S2 (Supplementary Materials).

The volatile profile of ‘Garden blue’ blueberry has been investigated previously, and the results showed that only part of the compounds played important roles in the blueberry aroma [23]. In fact, in this study, we also observed that most of the volatile compounds in blueberry were present at levels far below their sensory threshold. We did not observe any new compounds generated during the experimental period compared to the samples at harvest. Therefore, in this study, we mainly focused on the change of the volatile compounds in blueberry at the level higher or closer to their sensory thresholds (Figure 3). Nine volatile compounds were selected, including three esters (ethyl acetate, methyl isovalerate and ethyl 2-methylbutanoate), two C6 aldehydes (hexanal and E-2-hexenal), three monoterpenes (linalool, eucalyptol and α-terpineol), and one volatile phenol (eugenol).

The results show that ethyl acetate is the most abundant ester in ‘Garden blue’ blueberry. However, ethyl 2-methylbutanoate probably has higher aroma potency due to its low odor threshold. For the room temperature (no cold storage) treatment, the concentration of ethyl acetate significantly increased on the 4th day of storage at 25 °C, reached 10,064 μg/L on the 6th day, and then decreased quickly. Similar trends were also observed for ethyl acetate during the shelf life for the 15 and 60 d of cold storage treatments. Ethyl acetate reached its maximum level two days earlier than with no cold storage treatment. Without cold storage, methyl isovalerate content increased continually, with a large increment during the first two days. After 15 days of cold storage, the methyl isovalerate level also increased during the 25 °C storage period. However, after 60 days of cold storage, the methyl isovalerate level firstly increased then decreased during the subsequent 25 °C storage. A similar phenomenon was also observed for ethyl 2-methylbutanoate.

Hexanal and E-2-hexenal are two C6 aldehydes in the blueberry that mainly contribute to the green or leafy odor [21]. For the no cold storage treatment, the concentration of hexanal and E-2-hexenal decreased fast in the first 4 days, then hexanal started to increase, but the E-2-hexenal level became stable. After 15 and 60 days of cold storage, the concentrations of hexanal and E-2-hexenal were lower than in the fresh sample. The concentration of hexanal fluctuated, while E-2-hexenal decreased slowly during shelf life for both 15 and 60 d cold storage treatments.

Linalool and α-terpineol contribute to the floral notes of blueberry [23]. In this study, the concentration of α-terpineol was slightly below its sensory threshold (Figure 3). Considering that an aroma compound close to its threshold could also have sensory impact [31], we included α-terpineol in the quantification. Without cold storage, linalool content decreased from 0 to 4 d and increased from 4 to 8 d after harvest. After 15 and 60 days of cold storage, linalool content was significantly lower than the fresh sample. After cold storage, the linalool content in blueberry was relatively stable from 0 d to 4 d of subsequent shelf life and started to decrease afterward. Similar to linalool, α-terpineol content decreased from 0 d to 4 d and increased from 4 d to 8 d at 25 °C after harvest. For 15 d and 60 d cold storage treatments, α-terpineol showed a short increase during the first 2 days and decreased afterward during the subsequent shelf life. The aroma of eucalyptol has been described as ‘eucalyptus’, ‘fresh’, ‘cool’, ‘medicinal’, and ‘camphoraceous’ [32], and has been found to be closely related to the minty odor of blueberry [23]. The concentration of eucalyptol decreased quickly during the 25 °C storage and also decreased fast during the cold storage period. After 15 days and 60 days of cold storage, eucalyptol content in the blueberry decreased 76.9% and 86.8% respectively compared to its initial content at harvest (2.42 μg/kg).

### 3.5. Off-Odor

**Figure 4:**
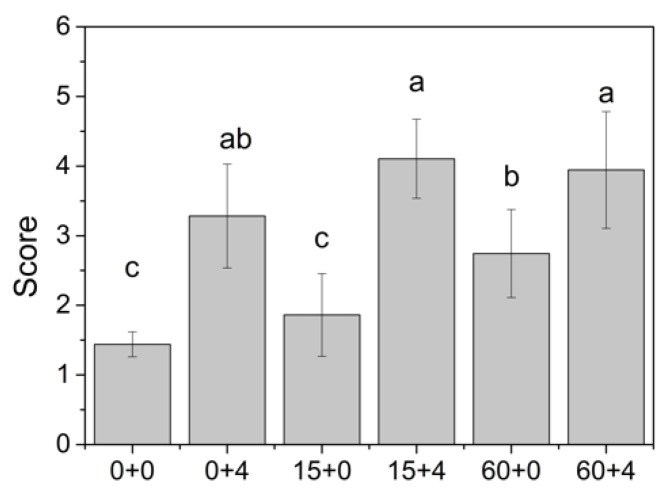
Sensory evaluation of off-flavor. 0 + 0: the blueberries prior to storage; 0 + 4: after storage at 25 °C for 4 days; 15 + 0: after storage at 0 °C for 15 days; 15 + 4: after storage at 0 °C for 15 days and subsequent storage at 25 °C for 4 days; 60 + 0: after storage at 0 °C for 60 days; 60 + 4: after storage at 0 °C for 60 days and subsequent storage at 25 °C for 4 days. Different letters indicate statistically significant difference (Tukey’s HSD, *p* < 0.05) between samples.

Off-flavor is defined as unpleasant odors or flavors imparted to food through internal deteriorative change [33]. In this study, off-flavor was defined as unpleasant odor that differed from the fresh samples. The results of sensory evaluation showed that the score of off-odor increased with increasing storage time (Figure 4). Off-odor developed faster under higher temperature storage conditions, and unpleasant odor became noticeable only after 4 days of storage at 25 °C. Cold storage could keep the blueberry odor at acceptable level (off-flavor score < 3) for over 60 days.

## 4. Discussion

‘Garden blue’ is a rabbiteye blueberry cultivar with high total soluble solids and good hardiness [34]. It is generally accepted that blueberry tends to soften during transportation and postharvest storage. However, in our study, increase of firmness was observed during storage. Berry firmness continually increased when the weight loss was below 2.55%. After 15 days of cold storage, the berry firmness still increased for 4 days until the weight loss increased to 5%, then the berry firmness started to decrease. Our results suggested that if the weight loss were well controlled, the firmness of blueberries could be well maintained at low temperature, and slightly increasing the storage temperature could increase the firmness of blueberries in the short term. Since the room temperature storage period was short compared to other studies, the expected berry softening was not obvious. Nevertheless, blueberry firming during storage has been observed by many researchers. For example, Chen et al. [23] reported that, irrespective of the storage temperatures, firmness of blueberries increased during the first 7 days of storage, and declined gradually afterwards. Chiabrando et al. [35] also reported that for blueberry cultivars ‘Bluecrop’ and ‘Coville’, berry firmness increased over 36% after storage at 0 °C for 5 weeks. Unfortunately, unlike the berry softening, the mechanism of berry firming has not been well explained yet. Paniagua et al. [36] pointed out that berry firming occurred consistently with low levels of weight loss (0.22–1.34%), whereas softening occurred with higher weight loss (3.47–15.06%). It was suggested that the firming of the blueberry during storage may be related to the parenchyma cell wall thickening, stone cell arrangement, and thickening of stone cell walls [37].

In recent decades, it has been generally accepted that the drop-off in flavor quality is one of the major sources of consumer dissatisfaction with fresh fruit products [38,39]. However, berry fruit flavor is delicate and hard to control during transportation and storage. Cold storage and transportation are the most widely used techniques for maintaining blueberry quality. Indeed, our results showed that esters in blueberries were relatively stable during cold storage for up to 60 days, but monoterpenes such as eucalyptol and linalool continually decreased. We also observed that the aroma compounds changed much more quickly under the subsequent higher temperature condition, indicating the flavor change could occur not only during the commercial storage and transportation process, but also during retail display or home storage. In fact, the aroma change under these conditions could be more noteworthy, since the changes occurring in just a few days depend on the temperature.

The change of blueberry odor during postharvest storage was probably due to the changes of esters, C6 aldehydes and terpenes. The most remarkable change was the concentration fluctuation of ethyl acetate. To better understand the possible sensory effect of these changes, the odor threshold of each compound was plotted with the concentration change in Figure 3. It has to be mentioned that the odor threshold of ethyl acetate was different in different studies. Therefore, we chose the most frequently reported threshold: 5000 μg/L in water [40]. In the fresh berries, the concentration of ethyl acetate was lower than its odor threshold. However, during 25 °C storage, the concentration of ethyl acetate increased to the level well above its threshold in just 4~6 days, then dropped quickly. Our observation was consistent with a previous report that the concentrations of esters were higher in overripe blueberries compared to ripe ones [16]. A similar phenomenon has also been observed in other fruits, such as over-ripened bananas and apples [41,42], and was associated with increased pyruvate decarboxylase activity [43]. The accumulated ethyl acetate during storage might impart an ‘over-ripe’ off-odor, and could result in a suppressive effect on the perception of other esters [44].

It has been reported that monoterpene formed in blueberry during the berry ripening stage [15], but little information was found regarding the monoterpene change during postharvest storage. In ‘Garden blue’, the linalool content was much higher than that of other monoterpenes, which was consistent with a previous report that linalool was an important compound contributing to the flavor of ‘Garden blue’ [23]. It has been reported in grapes that during storage, linalool can be lost by emission from the berries’ surfaces [45], which probably also occurs in blueberry, as the linalool content successively decreased after 15 d and 60 d of cold storage. Interestingly, the linalool content, which decreased during low-temperature storage, slightly increased during the subsequent storage at 25 °C. On the contrary, the eucalyptol content decreased rapidly during postharvest storage despite the temperature. Eucalyptol, which imparts a subtle minty aroma to blueberry, dropped to a level lower than its odor threshold in just four days. Minty aroma is a pleasant and distinct flavor for some blueberry cultivars, but seems hard to maintain during storage according to our observation.

The C6 aldehydes are generally considered to be products of fatty acid oxidation through the lipoxygenase pathway [46]. Like in cucumber and tomato, C6 aldehydes are important flavor compounds in blueberries, regardless of whether they are generated by oxidation [21,47]. Increase of C6 aldehydes is often observed in fruit during the postharvest storage [48], and has been found to be related to chilling injuries in some fruits such as in peaches and bananas [41,49]. However, in our study, cold storage did not result in increment of C6 aldehydes in “Garden blue” blueberry. This proves that cold storage is an effective way of preserving the quality of blueberries.

According to the sensory evaluation, prolonged storage did not negatively affect the flavor of ‘Garden blue’ blueberries. Extending postharvest storage from 15 to 60 days still resulted in an acceptable odor. However, subsequent storage at higher temperature resulted in a quick deterioration in sensory acceptability from “good” and “excellent” at harvest to “unpleasant off-odor” after 4 days. Due to the experimental design, we could not perform comparative descriptive analysis, since the samples had to be evaluated at different times. However, according to the panelists, the observed deterioration in blueberry flavor during storage was attributed to a slight decrease in sourness, a gradual decrease in typical blueberry flavor such as minty and floral odors, and enhanced accumulation of off-flavors described as ‘over-ripe’, ‘stale’, and ‘solvent-like’, which was consistent with the instrumental data.

## 5. Conclusions

Blueberry aroma is one of the most important factors affecting its quality. To simulate the storage conditions of blueberries in real production, we studied the changes of different quality parameters of blueberries during different length of cold storage conditions and subsequent room temperature storage, with special regard to volatile changes and ‘off-odor’ generation. The results proved that cold storage was a reliable way to preserve most of the quality traits of blueberry, as the firmness, pH, TSS and TA of blueberry changed slowly during cold storage. Aroma compounds generally showed a downward trend during the cold storage, but the decline was relatively slow. In contrast, the shelf life after cold storage is the most remarkable period of aroma change, which is represented by the strong fluctuation of ethyl acetate and the rapid decrease of terpenoids. Our results showed that aroma preservation during shelf life may be more important in the postharvest chain, and more research is still needed to better understand the mechanism behind the aroma change, and to explore ways to regulate the flavor of blueberries during shelf life.

## Figures and Tables

**Table 1:** Changes of pH, total soluble solids and firmness of blueberry stored at different temperatures.

0 °C Storage Time	Subsequent 25 °C Storage Time	pH	Total Soluble Solids (Brix)	TA (meq/L)	Firmness (N)
0 d	0 d	2.94 ± 0.2 ^b^	14.3 ± 0.6	6.52 ± 0.10 ^a^	4.08 ± 1.16 ^b^
	2 d	3.01 ± 0.1 ^ab^	13.9 ± 0.6	6.40 ± 0.22 ^ab^	4.45 ± 0.72 ^b^
	4 d	3.06 ± 0.2 ^ab^	14.0 ± 0.3	6.35 ± 0.16 ^ab^	4.68 ± 1.22 ^ab^
	6 d	3.07 ± 0.2 ^ab^	13.2 ± 0.8	6.37 ± 0.35 ^ab^	5.06 ± 1.13 ^ab^
	8 d	3.17 ± 0.1 ^a^	13.7 ± 0.3	6.21 ± 0.12 ^b^	5.58 ± 1.26 ^a^
15 d	0 d	3.04 ± 0.09 ^c^	13.5 ± 1.0 ^ab^	6.44 ± 0.23	4.00 ± 1.00 ^c^
	2 d	3.14 ± 0.04 ^abc^	14.5 ± 0.3 ^a^	6.47 ± 0.19	4.86 ± 1.42 ^b^
	4 d	3.20 ± 0.03 ^ab^	14.6 ± 0.3 ^a^	6.30 ± 0.22	7.06 ± 1.34 ^a^
	6 d	3.21 ± 0.05 ^a^	13.0 ± 0.8 ^b^	6.33 ± 0.14	5.96 ± 1.82 ^ab^
	8 d	3.14 ± 0.03 ^bc^	12.5 ± 0.4 ^b^	6.21 ± 0.54	6.95 ± 0.99 ^a^
60 d	0 d	3.21 ± 0.09 ^bc^	15.7 ± 0.5 ^a^	5.34 ± 0.24	5.83 ± 2.31 ^a^
	2 d	3.27 ± 0.03 ^abc^	15.0 ± 0.6 ^ab^	5.26 ± 0.33	3.79 ± 1.53 ^b^
	4 d	3.31 ± 0.05 ^ab^	13.7 ± 0.3 ^c^	5.39 ± 0.45	6.53 ± 2.82 ^a^
	6 d	3.36 ± 0.03 ^a^	13.6 ± 0.6 ^c^	5.45 ± 0.39	5.35 ± 1.88 ^ab^
	8 d	3.19 ± 0.03 ^c^	14.2 ± 0.3 ^bc^	5.54 ± 0.47	6.10 ± 1.30 ^a^

Different letters in same column means there is statistically significant difference (Tukey’s HSD, *p* < 0.05) between samples.

## Supplementary Materials

The following are available online at https://www.mdpi.com/2304-8158/9/9/1223/s1, Figure S1: A representative chromatograph of ‘Garden blue’ blueberry volatiles detected by SPME-GC-QTOF-MS. The peak number is corresponding to the compound list in Table S1, Table S1: Quantification information for GC-QTOF-MS analysis, Table S2: Odor threshold and description of volatile compounds.

## References

[1] Howard L.R., Prior R.L., Liyanage R., Lay J.O. (2012). Processing and Storage Effect on Berry Polyphenols: Challenges and Implications for Bioactive Properties. J. Agric. Food Chem..

[2] Sater H.M., Bizzio L.N., Tieman D.M., Muñoz P.D. (2020). A Review of the Fruit Volatiles Found in Blueberry and Other Vaccinium Species. J. Agric. Food Chem..

[3] Caleb O.J., Mahajan P.V., Al-Said A.J., Opara U.L. (2013). Modified Atmosphere Packaging Technology of Fresh and Fresh-cut Produce and the Microbial Consequences—A Review. Food Bioprocess Technol..

[4] Wang C., Gao Y., Tao Y., Wu X. (2017). Influence of γ-irradiation on the reactive-oxygen metabolism of blueberry fruit during cold storage. Innov. Food Sci. Emerg..

[5] Bounous G., Giacalone G., Guarinoni A., Peano C. (1997). Modified atmosphere storage of high bush blueberry. Acta Hortic..

[6] Almenar E., Samsudin H., Auras R., Harte B., Rubino M. (2008). Postharvest shelf life extension of blueberries using a biodegradable package. Food Chem..

[7] Reque P.M., Steffens R.S., Jablonski A., Flôres S.H., Rios A.D.O., De Jong E.V. (2014). Cold storage of blueberry (*Vaccinium* spp.) fruits and juice: Anthocyanin stability and antioxidant activity. J. Food Compos. Anal..

[8] Villarreal N.M., Bustamante C.A., Civello P.M., Martinez G.A. (2010). Effect of ethylene and 1-MCP treatments on strawberry fruit ripening. J. Sci. Food Agric..

[9] Duan W., Sun P., Chen L., Gao S., Shao W., Li J. (2018). Comparative analysis of fruit volatiles and related gene expression between the wild strawberry *Fragaria pentaphylla* and cultivated *Fragaria* × *ananassa*. Eur. Food Res. Technol..

[10] Maclean D., Nesmith D.S. (2011). Rabbiteye Blueberry Postharvest Fruit Quality and Stimulation of Ethylene Production by 1-Methylcyclopropene. Hortscience.

[11] Fu X.M., Cheng S.H., Zhang Y.Q., Du B., Feng C., Zhou Y., Mei X., Jiang Y.M., Duan X.W., Yang Z.Y. (2017). Differential responses of four biosynthetic pathways of aroma compounds in postharvest strawberry (*Fragaria* × *ananassa* Duch.) under interaction of light and temperature. Food Chem..

[12] Forney C.F. (2001). Horticultural and other Factors Affecting Aroma Volatile Composition of Small Fruit. HortTechnology.

[13] Dymerski T., Namieśnik J., Vearasilp K., Arancibia-Avila P., Toledo F., Weisz M., Katrich E., Gorinstein S. (2015). Comprehensive two-dimensional gas chromatography and three-dimensional fluorometry for detection of volatile and bioactive substances in some berries. Talanta.

[14] Horvat R.J., Senter S.D., Dekazos E.D. (1983). GLC-MS Analysis of Volatile Constituents in Rabbiteye Blueberries. J. Food Sci..

[15] Horvat R.J., Schlotzhauer W.S., Chortyk O.T., Nottingham S.F., Payne J.A. (1996). Comparison of Volatile Compounds from Rabbiteye Blueberry (*Vaccinium ashei*) and Deerberry (*V. stamineum*) during Maturation. J. Essent. Oil Res..

[16] Farneti B., Khomenko I., Grisenti M., Ajelli M., Betta E., Algarra A.A., Cappellin L., Aprea E., Gasperi F., Biasioli F. (2017). Exploring blueberry aroma complexity by chromatographic and direct-injection spectrometric techniques. Front. Plant Sci..

[17] Liu F., Li S., Gao J., Cheng K., Yuan F. (2019). Changes of terpenoids and other volatiles during alcoholic fermentation of blueberry wines made from two southern highbush cultivars. LWT Food Sci. Technol..

[18] Yuan F., Cheng K., Gao J., Pan S. (2018). Characterization of Cultivar Differences of Blueberry Wines Using GC-QTOF-MS and Metabolic Profiling Methods. Molecules.

[19] Zhu N., Zhu Y., Yu N., Wei Y., Zhang J., Hou Y., Sun A.D. (2018). Evaluation of microbial, physicochemical parameters and flavor of blueberry juice after microchip-pulsed electric field. Food Chem..

[20] Gilbert J.L., Schwieterman M.L., Colquhoun T.A., Clark D.G., Olmstead J.W. (2013). Potential for Increasing Southern Highbush Blueberry Flavor Acceptance by Breeding for Major Volatile Components. Hortscience.

[21] Du X., Rouseff R. (2014). Aroma active volatiles in four southern highbush blueberry cultivars determined by gas chromatography–olfactometry (GC-O) and gas chromatography–mass spectrometry (GC-MS). J. Agric. Food Chem..

[22] Ferrao L.F.V., Johnson T.S., Benevenuto J., Edger P.P., Colquhoun T.A., Munoz P.R. (2020). Genome-wide association of volatiles reveals candidate loci for blueberry flavor. New Phytol..

[23] Cheng K., Peng B.Z., Yuan F. (2020). Volatile composition of eight blueberry cultivars and their relationship with sensory attributes. Flavour Fragr. J..

[24] Yang J., Shi W., Li B., Bai Y., Hou Z. (2019). Preharvest and postharvest uv radiation affected flavonoid metabolism and antioxidant capacity differently in developing blueberries (*Vaccinium corymbosum* L.). Food Chem..

[25] Li X., Jin L., Pan X., Yang L., Guo W. (2019). Proteins expression and metabolite profile insight into phenolic biosynthesis during highbush blueberry fruit maturation. Food Chem..

[26] Eichholz I., Huyskens-Keil S., Keller A., Ulrich D., Kroh L.W., Rohn S. (2011). UV-B-induced changes of volatile metabolites and phenolic compounds in blueberries (*Vaccinium corymbosum* L.). Food Chem..

[27] Cao S., Hu Z., Pang B., Wang H., Xie H., Wu F. (2010). Effect of ultrasound treatment on fruit decay and quality maintenance in strawberry after harvest. Food Control.

[28] Lin Y., Huang G., Zhang Q., Wang Y., Meng X. (2020). Ripening affects the physicochemical properties, phytochemicals and antioxidant capacities of two blueberry cultivars. Postharvest Biol. Technol..

[29] Paniagua A.C., East A.R., Heyes J.A. (2013). Effects of delays in cooling on blueberry quality outcomes. Acta Hortic..

[30] Sanford K.A., Lidster P.D., Mcrae K.B., Jackson E.D., Lawrence R.A., Stark R., Prange R.K. (1991). Lowbush blueberry quality changes in response to mechanical damage and storage temperature. J. Am. Soc. Hortic. Sci..

[31] Ribereau-Gayon P., Boidron J.N., Terrier A. (1975). Aroma of Muscat grape varieties. J. Agric. Food Chem..

[32] Capone D.L., Van Leeuwen K., Taylor D.K., Jeffery D.W., Pardon K.H., Elsey G.M., Sefton M.A. (2011). Evolution and occurrence of 1,8-cineole (eucalyptol) in australian wine. J. Agric. Food Chem..

[33] Kilcast D., Baigrie B. (2003). Taints and Off-Flavours in Foods.

[34] Dozier W.A., Caylor A.W., Himelrick D.G., Powell A.A., Akridge J.R. (1991). Rabbiteye blueberry cultivar performance. Fruit Var. J..

[35] Chiabrando V., Giacalone G., Rolle L. (2009). Mechanical behaviour and quality traits of highbush blueberry during postharvest storage. J. Sci. Food Agric..

[36] Paniagua A.C., East A.R., Hindmarsh J.P., Heyes J. (2013). Moisture loss is the major cause of firmness change during postharvest storage of blueberry. Postharvest Biol. Technol..

[37] Allanwojtas P.M., Forney C.F., Carbyn S.E., Nicholas K.U. (2001). Microstructural Indicators of Quality-related Characteristics of Blueberries—An Integrated Approach. LWT Food Sci. Technol..

[38] Alvarez M.V., Ponce A.G., Moreira M.R. (2018). Influence of polysaccharide-based edible coatings as carriers of prebiotic fibers on quality attributes of ready-to-eat fresh blueberries. J. Sci. Food Agric..

[39] Klee H.J. (2010). Improving the flavor of fresh fruits: Genomics, biochemistry, and biotechnology. New Phytol..

[40] van Gemert L.J. (2011). Odour Thresholds. Compilations of Odour Threshold Values in Air, Water and Other Media.

[41] Dixon J., Hewett E.W. (2000). Factors affecting apple aroma/flavour volatile concentration: A review. N. Z. J. Crop Hortic. Sci..

[42] Zhu X., Luo J., Li Q., Li J., Liu T., Wang R., Chen W., Li X. (2018). Low temperature storage reduces aroma-related volatiles production during shelf-life of banana fruit mainly by regulating key genes involved in volatile biosynthetic pathways. Postharvest Biol. Technol..

[43] Yuan F., Yan J., Yan X., Liu H., Pan S. (2020). Comparative transcriptome analysis of genes involved in volatile compound synthesis in blueberries (*Vaccinium virgatum*) during postharvest storage. Postharvest Biol. Technol..

[44] Francis I., Newton J. (2005). Determining wine aroma from compositional data. Aust. J. Grape Wine Res..

[45] Matsumoto H., Ikoma Y. (2016). Effect of postharvest temperature on the muscat flavor and aroma volatile content in the berries of ‘Shine Muscat’ (*Vitis labruscana* Baily × *V. vinifera* L.). Postharvest Biol. Technol..

[46] Siegmund B. (2015). Flavour Development Analysis & Perception in Food & Beverages.

[47] Beaulieu J.C., Stein-Chisholm R.E., Boykin D.L. (2014). Qualitative Analysis of Volatiles in Rabbiteye Blueberry Cultivars at Various Maturities Using Rapid Solid-phase Microextraction. J. Am. Soc. Hortic. Sci..

[48] Krumbein A., Peters P., Bruckner B. (2004). Flavour compounds and a quantitative descriptive analysis of tomatoes (*Lycopersicon esculentum* Mill.) of different cultivars in short-term storage. Postharvest Biol. Technol..

[49] Brovelli E.A., Brecht J.K., Sherman W.B., Sims C.A. (1998). Quality of Fresh-Market Melting- and Nonmelting-Flesh Peach Genotypes as Affected by Postharvest Chilling. J. Food Sci..

