# Supplementary material for: Changes of the Aroma Composition and Other Quality Traits of Blueberry ‘Garden Blue’ during the Cold Storage and Subsequent Shelf Life
Source: Foods. 2020 Sep 2;9(9):1223. doi: 10.3390/foods9091223 (PMC7555369; doi:10.3390/foods9091223)
Supplement: Supplementary file 1 [file foods-09-01223-s001.pdf]

Supplementary Table and Figure

Table S1. Quantification information for GC-QTOF-MS analysis

Peak No.	RT	Compound	Quantify ion	Regression equation	R2
1	14.80	4-octanol (IS)	55.0521		
2	2.18	ethyl acetate	70.0393	y=0.046588*x	0.9560
3	4.99	methyl isovalerate	74.0342	y=0.5615*x	0.9819
4	5.76	hexanal	56.0607	y=0.1836*x	0.9939
5	7.89	ethyl 2-methylbutanoate	102.0655	y=1.1273*x	0.9857
6	7.90	E-2-hexenal	98.0710	y=0.0659*x^2+0.0067*x	0.9898
7	16.85	eucalyptol	81.0682	y=0.8529*x	0.9974
8	20.41	linalool	93.0671	y=1.0808*x	0.9959
9	24.76	α-terpineol	93.0671	y=0.7229*x	0.9858
10	32.40	eugenol	164.0805	y=0.0485*x^2+0.0634*x	0.9974

$x = A_{st}/A_{is} - A_{matrix}/A_{is\ in\ matrix}$ and $y = C_{st}/C_{is}$ with A_{st} = area of standard, A_{is} = areas of internal standard, A_{matrix} = area peak in matrix, $A_{is\ in\ matrix}$ = areas of internal standard in matrix, C_{st} = concentration of standard, and C_{is} = concentration of internal standard. 4-Octanol was used as internal standard.

Table S2. Odor threshold and description of volatile compounds

Compound	Odor threshold	Odor description
ethyl acetate	5000	Ethereal, fruity-grape, sweet, rum-like
methyl isovalerate	4.4	Apple, fruity, pineapple
hexanal	0.1	Fresh, green, fatty, aldehydic, sweaty
ethyl 2-methylbutanoate	4.5	Cooked apple, apricot, orange, grapefruit
E-2-hexenal	17	Fresh, green
eucalyptol	1.3	Light Camphor alike odor
linalool	86	Fresh, floral-woody, sweet, citrus
α-terpineol	6	Floral lilac smell
eugenol	0.71	Sweet, spicy-clove, woody, dry, with phenolic, cinnamon and allspice nuances

The odor thresholds (μg/L in water) were obtained from literature: van Gemert, L.J. Odour thresholds. Compilations of odour threshold values in air, water and other media.; Oliemans, Punter & Partners BV: Utrecht, The Netherlands, 2011.

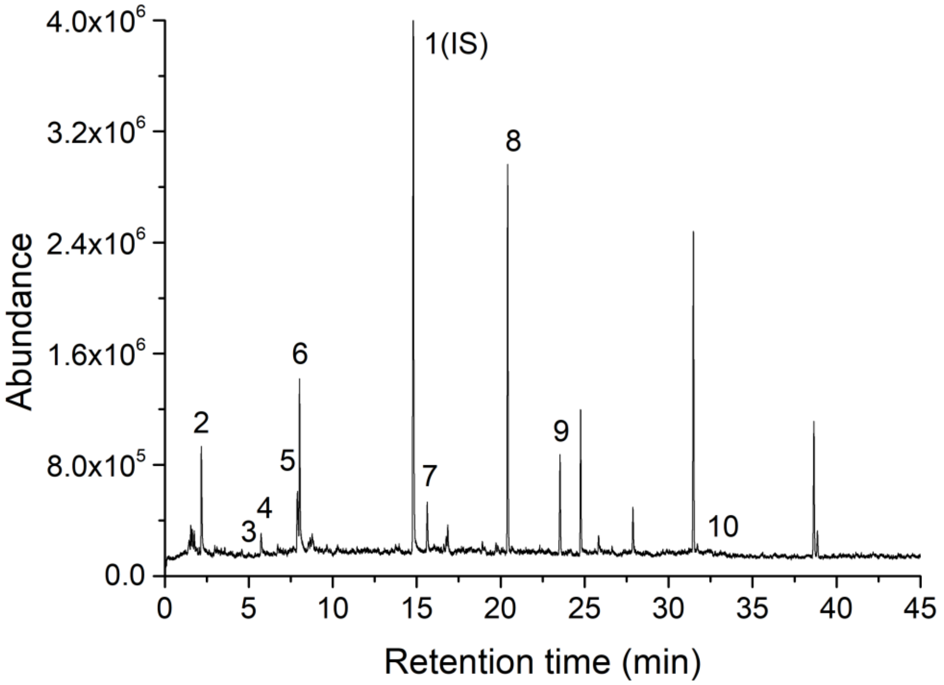

Figure S1. A representative chromatograph of 'Garden blue' blueberry volatiles detected by SPME-GC-QTOF-MS. The peak number is corresponding to the compound list in Table S1.
